# The use of intravenous immunoglobulin gamma for the treatment of severe coronavirus disease 2019: a randomized placebo-controlled double-blind clinical trial

**DOI:** 10.1186/s12879-020-05507-4

**Published:** 2020-10-21

**Authors:** Naser Gharebaghi, Rahim Nejadrahim, Seyed Jalil Mousavi, Seyyed-Reza Sadat-Ebrahimi, Reza Hajizadeh

**Affiliations:** 1 Department of Infectious Diseases, Urmia University of Medical Sciences, Urmia, Iran; 2 Cardiovascular Research Center, Tabriz University of Medical Sciences, Tabriz, Iran; 3 Department of Cardiology, Urmia University of Medical Sciences, Urmia, Iran

**Keywords:** Coronavirus disease 2019, COVID-19, SARS-CoV-2, Severe infection, Intravenous immunoglobulin

## Abstract

**Background:**

Coronavirus disease 2019 (COVID-19) has infected people in many countries worldwide. Discovering an effective treatment for this disease, particularly in severe cases, has become the subject of intense scientific investigation. Therefore, the objective of this study was to evaluate the efficacy of intravenous immunoglobulin (IVIg) in patients with severe COVID-19 infection.

**Methods:**

This study was conducted as a randomized placebo-controlled double-blind clinical trial. Fifty-nine patients with severe COVID-19 infection who did not respond to initial treatments were randomly assigned into two groups. One group received IVIg (human)—four vials daily for 3 days (in addition to initial treatment), while the other group received a placebo. Patients’ demographic, clinical, and select laboratory test results, as well as the occurrence of in-hospital mortality, were recorded.

**Results:**

Among total study subjects, 30 patients received IVIg and 29 patients received a placebo. Demographics, clinical characteristics, and laboratory tests were not statistically different (*P* > 0.05) between the two groups. The in-hospital mortality rate was significantly lower in the IVIg group compared to the control group (6 [20.0%] vs. 14 [48.3%], respectively; *P* = 0.022). Multivariate regression analysis demonstrated that administration of IVIg did indeed have a significant impact on mortality rate (aOR = 0.003 [95% CI: 0.001–0.815]; *P* = 0.042).

**Conclusions:**

Our study demonstrated that the administration of IVIg in patients with severe COVID-19 infection who did not respond to initial treatment could improve their clinical outcome and significantly reduce mortality rate. Further multicenter studies with larger sample sizes are nonetheless required to confirm the appropriateness of this medication as a standard treatment.

**Trial registration:**

A study protocol was registered at the Iranian Registry of Clinical Trials (www.IRCT.ir), number IRCT20200501047259N1. It was registered retrospectively on May 17th, 2020.

**Supplementary information:**

**Supplementary information** accompanies this paper at 10.1186/s12879-020-05507-4.

## Background

Coronavirus disease 2019 (COVID-19) was declared to be a pandemic by the World Health Organization on March 11th, 2020 [1]. The culprit virus, severe acute respiratory syndrome coronavirus 2 (SARS-CoV-2) is highly communicable and can spread through respiratory droplets [2]. Among Chinese patients, an overall mortality rate of 3·6% (95% CI 3·5–3·7) for COVID-19 has been reported [3]. Jalili et al. by studying 28,981 hospitalized COVID-19 patients in Iran showed that cumulative risk for death in 30 days was 24.4 (23.8–25.0 95% CI), ranging from 14.8 (14.2–15.4) in < 65 years old patients to 41.6(40.5–42.8) in those with ≥65 years old [4]. Lopinavir-Ritonavir, chloroquine phosphate, hydroxychloroquine, and alpha-interferon are thus far the most commonly used medications for COVID-19 [5]. Preclinical studies have proposed the use of Remdesivir (an RNA polymerase inhibitor with in vitro activity against multiple RNA viruses, including Ebola) and Tocilizumab (a humanized IgG1 monoclonal antibody, directed against the IL-6 receptor) [6]. The data about chloroquine is controversial, but completed studies are relatively in favor of its efficacy in the treatment of COVID-19 [7]. Although some studies support the use of Lopinavir-Ritonavir, Cao et al. demonstrated no obvious efficacy of Lopinavir-Ritonavir in COVID-19 treatment compared to standard treatment [8]. Alpha-interferon is recommended to use only in clinical trials [9]. Beigel et al. showed that Remdesivir was superior to placebo in shortening the duration of disease [9].

Currently, no vaccine for COVID-19 is widely available for the general population. It is estimated that it would be available by early 2021. About 78 projects have been developed for vaccine production, mostly by private industries. Although, the proposed mechanism of action of these vaccines varies greatly among the ongoing projects, using neutralizing antibodies against the viral spikes and its S protein is the main goal of most of them [10].

Intravenous immunoglobulin (IVIg) is a blood product that is obtained from healthy donors and contains polyclonal immunoglobulin gamma. Since its discovery as an effective treatment 30 years ago, it has been administered as an immunomodulatory therapy in autoimmune and inflammatory diseases such as immune thrombocytopenic purpura, Kawasaki disease, chronic inflammatory demyelinating polyneuropathy, and multifocal motor neuropathy [11]. Significant positive outcomes have been observed by the administration of IVIg in patients with SARS and Middle East respiratory syndrome (MERS) [12–14]. Considering the presence of an overwhelming immune response among many COVID-19 patients [15, 16], as well as similarities in pathogenesis between severe acute respiratory syndrome (SARS) and COVID-19, it seems feasible that IVIg may improve passive immunity and modulate inflammatory response in COVID-19 patients [17]. A recent case report in China described a significant clinical improvement in three patients with severe COVID-19 who received high dose IVIg [17]. The lack of broader research remains an impediment to proposing this treatment as a first-line therapeutic option against COVID-19.

## Methods

This study was a randomized double-blind placebo-controlled clinical trial (*n* = 59) among patients with severe COVID-19 who did not respond to initial treatments. The research was conducted in accordance with the Declaration of Helsinki protocol. Informed consent was obtained from patients or their guardians. The study protocol was approved by the medical ethics committee of the Urmia University of Medical Sciences (IR.UMSU.REC.1399.025), and was registered at the Iranian Registry of Clinical Trials with the registration number IRCT20200501047259N1 (www.IRCT.ir).

### Study sample

Patients were included if they had acute respiratory syndrome and a definitive diagnosis of COVID-19, made based on real-time reverse transcription-polymerase chain reaction (RT-PCR) and chest computed tomography scan findings from an undisclosed teaching hospital. The name of the teaching hospital was hidden due to blind peer-review protocol. Patients consecutively were recruited between May 9th, 2020 and June 9th, 2020. Inclusion criteria included being over 18 years of age, possessing a PCR-confirmed COVID-19 diagnosis, involvement of > than 30% of both lungs (ground-glass opacity) in high-resolution computed tomography (HRCT) (confirmed by two radiologists), O_2_ saturation (satO_2_) of < 90%, and a lack of adequate response to initial treatment including at least both one antiviral and one chloroquine-class drug. Exclusion criteria, in addition to an age of less than 18 years, included pregnancy, coagulation disorders (such as hemophilia, Von Willebrand disease, other clotting factor deficiencies), history of hypersensitivity to IVIg, advanced heart failure (defined as a left ventricular ejection fraction less than 35%), pulmonary fibrosis/history of lung surgery, and the presence of either sarcoidosis or tuberculosis (that may interfere with an accurate estimation of the severity of pulmonary interference by COVID-19).

Inadequate response to initial treatment was defined as the lack of improvement of dyspnea, fever, *and* hypoxemia (satO_2_ less than 90%), as well as the need for oxygenation to maintain satO_2_ above 90% after 48 h of commencing treatment.

### Exposure

Study subjects were randomly assigned into two approximately equal groups: IVIg treatment and placebo control using a computer-generated randomization schedule. The IVIg group received IVIg (human) flebogamma 5% DIF GRIFOLS, in addition to their prior initial treatment (the initial treatment methods continued in the treatment group during the trial). Treatment group patients received four vials of 5 gm5 IVIg daily for three consecutive days. Those patients who died before 72 h after the distribution of IVIg and placebos were excluded from our study due to an incomplete course of treatment. The control group continued to receive the same treatments as were introduced initially, in addition to a placebo. Neither patients nor physicians nor data analysts were aware of treatment versus placebo membership. The only individual that did was the pharmacist of the study center. Placebo and IVIg vials were similar in appearance and contained a similar volume of solution. Placebo vials contained saline solution.

### Data analysis

Normal distribution of all continuous variables was evaluated using a Kolmogorov-Smirnov test. The majority of continuous variables (both the demographic and clinical variables) did not possess a normal distribution; therefore, the median and interquartile range (IQR, 25th percentile – 75th percentile) of all continuous variables was reported. The frequency and percentage of categorical variables” were reported. Continuous variables were compared using a Mann-Whitney U test. Categorical variables were compared using a chi-square or Fisher exact test. Univariate logistic regression was used to model mortality rates based on all investigated variables. Statistically significant variables (both the demographic and clinical variables) in univariable logistic regression (significance is evaluated at the 0.2 alpha level) were entered into multivariable logistic regression. All statistical analyses were conducted using SPSS version 22 (SPSS Inc., Chicago, IL) [18].

## Results

The investigated characteristics of the subjects are described in Table 1. Demographics, clinical characteristics, and evaluated laboratory tests between the treatment and control group did not exhibit significant differences except for between: (1) serum creatinine (mg/dl), which was higher in the control group (1.0 [0.8–1.1] in the treatment group vs. 1.2 [1.0–1.4] in the control group; *P* = 0.001), (2) white blood cell (WBC) count (1000/mm^3^), which was also higher in the control group (5.05 [4.20–7.00] in the treatment group vs. 6.60 [5.00–10.90] in the control group; *P* = 0.026), and (3) the overall duration of hospitalization (days) was longer in the treatment group (9 [7–13] in the treatment group vs. 7 [6–9] in the control group; *P* = 0.014).

**Table 1:** Evaluated characteristics of patients with severe COVID-19 infection

	Total	IVIg Group	Control Group	***P***-value^**$**^
**Age (years)**^**a**^	56 (46,62)	55.5 (45,60)	56 (47,66)	0.375
**Sex n (%)**				
**Male**	41 (69.5)	21 (70)	20 (68.9)	0.931
**Female**	18 (30.5)	9 (30)	9 (31)	
**HTN n (%)**	13 (22)	7 (23.3)	6 (20.6)	0.807
**DM n (%)**	16 (27.1)	6 (20)	10 (34.4)	0.211
**Chronic lung disease n (%)**	2 (3.3)	2 (6.6)	0 (0)	0.157
**HR/min**	95 (89,105)	92.5 (89,100)	96 (90,108)	0.280
**Systolic BP (mmHg)**	120 (115,130)	120 (120,130)	120 (110,130)	0.428
**Diastolic BP (mmHg)**	80 (70,80)	80 (70,80)	80 (70,80)	0.542
**RR /min**	19 (18,22)	19.5 (18,22)	19 (18,21)	0.927
**BT (°C)**	37.1 (36.7,37.7)	37.05 (36.5,37.8)	37.1 (36.9,37.6)	0.772
O_2_ **saturation (%)**	88 (85,89)	88 (85,89)	88 (85,89)	0.436
**WBC (1000/mm**^**3**^**)**	5.6 (4.6,8.7)	5.0 (4.2,7)	6.6 (5,10.9)	**0.026**
**Neutrophil (%)**	78 (70,83)	74 (70,80)	80 (74,87)	0.114
**Lymphocyte (%)**	18 (11,22)	19 (14,25)	16 (9,20)	0.085
**Hb (g/dl)**	13.9 (12.4,15)	13.7 (12.2,15)	14 (13.1,15.1)	0.309
**Plt (1000/mm**^**3**^**)**	190 (137,226)	186 (133,220)	191 (160,234)	0.457
**LDH (U/L)**	591 (444,742)	545.5 (473,705)	611 (421,800)	0.677
**BUN (mg/dl)**	34 (27,58)	30.5 (27,46)	50 (27,68)	0.082
**Creatinine (mg/dl)**	1.1 (1,1.3)	1 (0.8,1.1)	1.2 (1,1.4)	**0.001**
**K (mEq/L)**	4.1 (3.9,4.4)	4 (3.9,4.5)	4.1 (3.9,4.3)	0.813
**Na (mEq/L)**	138 (136,140)	138 (137,140)	138 (135,143)	0.728
**ESR**	29 (20,46)	28 (23,50)	31 (20,41)	0.808
**AST (U/L)**	31 (21,42)	34.5 (21,53)	29 (18,40)	0.271
**ALT (U/L)**	35 (27,45)	34 (27,42)	38 (24,47)	0.596
**BS (mg/dl)**	120 (106,174)	118 (105,141)	131 (109,229)	0.295
**pH**	7.3 (7.3,7.4)	7.4 (7.3,7.4)	7.3 (7.3,7.4)	0.210
**Pa**O_2_ **(mmHg)**	45 (38,49)	45 (40,49)	45 (37,50)	0.767
**PC**O_2_ **(mmHg)**	39 (36,45)	38 (35,42)	39 (38,47)	0.084
**HCO**_**3**_ **(mEq/L)**	24 (21,26)	24 (23,26)	24 (21,26)	0.522
**Duration of stay in ICU (days)**	3 (2,6)	4 (3,6)	3 (2,4)	0.101
**Duration of hospitalization (days)**	8 (6,11)	9 (7,13)	7 (6,9)	**0.014**

*IVIg* Intravenous immunoglobulin, *HR* Heart rate, *BP* Blood pressure, *RR* Respiratory rate, *BT* Body temperature, *WBC* White blood cells, *HB* Hemoglobin, *PLT* Platelet, *LDH* Lactate dehydrogenase, *BUN* Blood urea nitrogen, *K* Serum potassium, *Na* Serum sodium, *ESR* Erythrocyte sedimentation rate, *AST* Enzymes aspartate transaminase, *ALT* alanine aminotransferase, *ALP* Alkaline phosphatase, *FBS* Fasting blood sugar, *BS* Blood sugar, *PaO*_*2*_ Partial pressure of oxygen, *PCO*_*2*_ Partial pressure of carbon dioxide, *HCO3* Bicarbonate, *ICU* Intensive care unit

^a^ Data are presented using median (and IQR), except for the following categories (reported as frequency and percentage): sex, HTN, DM, chronic lung disease. ^$^ Comparison between IVIg and control groups

**Fig. 1:**
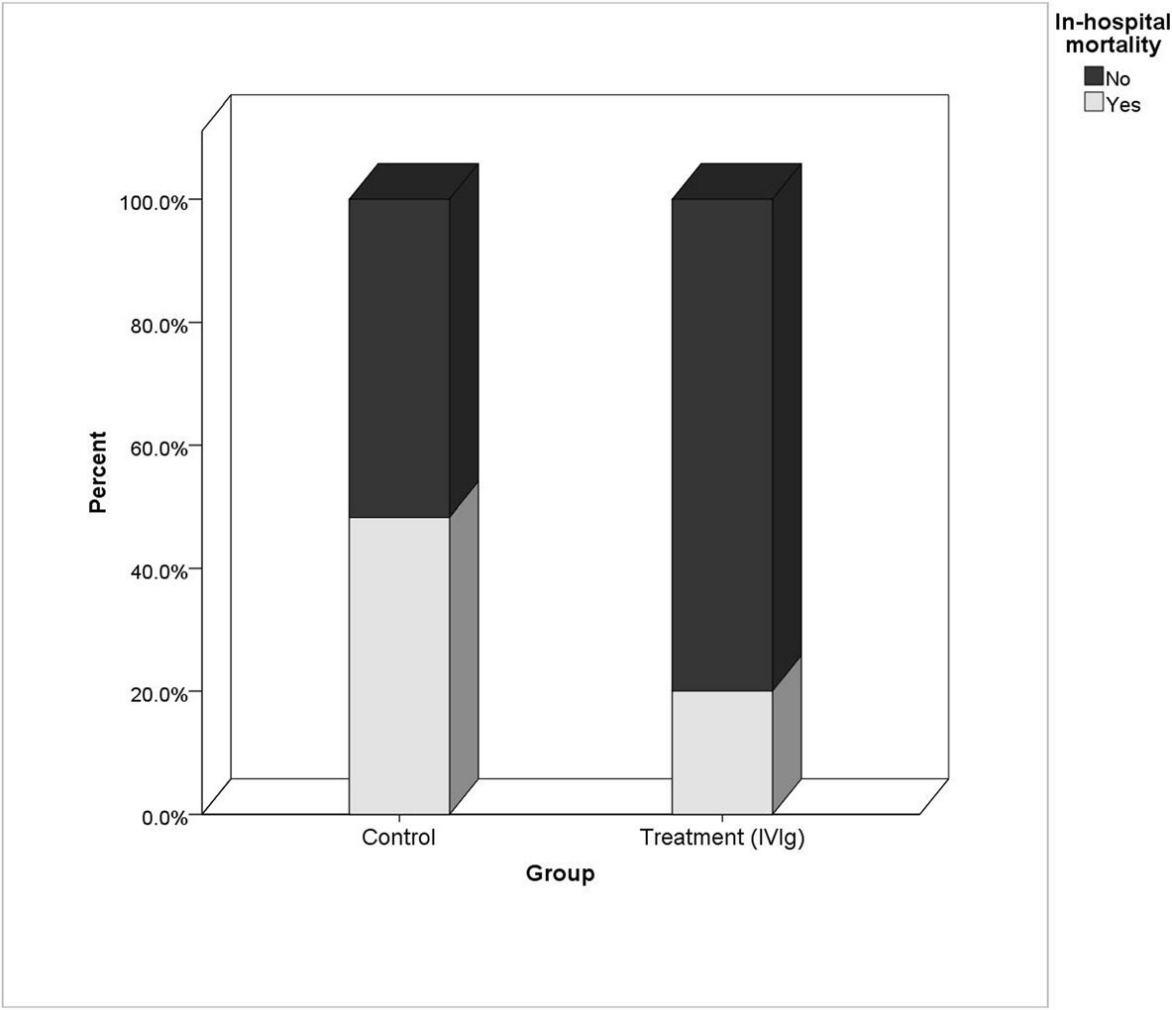
In-hospital mortality rate in treatment and control groups

The in-hospital mortality rate was significantly lower in the treatment group (6 [20.0%] in the treatment group vs 14 [48.3%] in the control group; *P* = 0.025; Table 2, Fig. 1). Univariate regression analysis identified several variables potentially related to the mortality of patients (Table 2). By adjusting these variables, multivariate regression analysis demonstrated that the administration of IVIg had a statistically significant impact on in-hospital mortality and was thus an independent determinant of mortality (aOR = 0.003 [0.001–0.815]; *P* = 0.042). Moreover, increasing age, lower diastolic blood pressure, and increasing serum lactate dehydrogenase (LDH) were other determinants of elevated mortality in patients with severe COVID-19 infection (Table 3).

**Table 2:** The relationship between study variables and mortality of patients with severe COVID-19

	Mortality		Unadjusted OR (95% CI)	***P***-value
	No	Yes		
**Groups**				
**Treatment**	15 (38.4)	14 (70)	0.27 (0.08, 0.85)	**0.025**
**Control**	24 (61.5)	6 (30)	Ref	
**Age (years)**	54 (44, 60)	60 (53.5, 70)	1.05 (1.01, 1.10)	**0.014**
**Gender n (%)**				
**Male**	27 (70)	14 (70)	0.96 (0.30, 3.12)	0.951
**Female**	12 (30)	6 (30)	Ref	
**HTN n (%)**	8 (23.3)	5 (25)	1.29 (0.36, 4.63)	0.694
**DM n (%)**	11 (20)	5 (25)	0.85 (0.25, 2.90)	0.793
**Chronic lung disease n (%)**	1 (6.6)	1 (5)	2.00 (0.12, 33.76)	0.630
**HR/min**	95 (90, 100)	95 (89, 108)	1.02 (0.96, 1.08)	0.543
**Systolic BP (mmHg)**	120 (120, 130)	120 (110, 130)	0.97 (0.93, 1.01)	**0.173**
**Diastolic BP (mmHg)**	80 (70, 80)	70 (70, 80)	0.95 (0.88, 1.01)	**0.120**
**RR /min**	19 (18, 22)	20 (18, 22)	0.98 (0.93, 1.04)	0.545
**BT (C°)**	37.1 (36.7, 37.7)	37.1 (36.75, 37.65)	1.26 (0.63, 2.52)	0.518
O_2_ **saturation (%)**	89 (87, 89)	85 (80, 88)	0.84 (0.74, 0.97)	**0.015**
**WBC (1000/mm**^**3**^**)**	5.1 (4.6, 7.4)	7.2 (5.1, 11.2)	1.00 (1.00, 1.00)	0.743
**HB (g/dl)**	13.9 (12.6, 15)	13.5 (12.05, 15.25)	0.95 (0.80, 1.13)	0.566
**PLT(1000/mm**^**3**^**)**	210 (135, 247)	172.5 (139.5, 193)	1.00 (1.00, 1.00)	**0.090**
**LDH (U/L)**	520 (400, 687)	761 (560.5, 1021)	1.00 (1.00, 1.00)	**0.003**
**BUN (mg/dl)**	29 (25, 39)	64 (50, 129.5)	1.06 (1.02, 1.09)	**0.001**
**Creatinine (mg/dl)**	1 (1, 1.2)	1.2 (1.1, 1.55)	3.63 (0.95, 13.87)	**0.059**
**K (mEq/L)**	4 (3.7, 4.4)	4.2 (4.1, 4.3)	0.95 (0.80, 1.14)	0.616
**Na (mEq/L)**	138 (137, 139)	138 (135, 143.5)	1.04 (0.95, 1.14)	0.344
**ESR**	28 (20, 41)	40 (26, 49.5)	1.01 (0.99, 1.03)	0.424
**AST (U/L)**	31 (21, 39)	29.5 (15.5, 52.5)	1.00 (0.97, 1.02)	0.735
**ALT (U/L)**	34.5 (25, 41)	38.5 (29.5, 48)	1.01 (0.99, 1.02)	0.306
**BS (mg/dl)**	119 (105, 174)	126 (109.5, 184)	1.00 (0.99, 1.01)	0.845
**Pa**O_2_ **(mmHg)**	46 (40, 50)	39.5 (34, 49)	0.92 (0.85, 0.99)	**0.035**
**PC**O_2_ **(mmHg)**	40 (35, 46)	38 (36, 39.5)	0.99 (0.91, 1.08)	0.798
**HCO**_**3**_ **(mEq/L)**	24 (22, 25)	24.5 (19.5, 26.5)	1.00 (0.89, 1.13)	0.946
**pH**	7.3 (7.3, 7.4)	7.3 (7.2, 7.4)	0.04 (0.00, 8.87)	0.243

Variables with *P*-value less than 0.2 were selected to enter multivariable regression analysis

*IVIg* Intravenous immunoglobulin, *HR* Heart rate, *BP* Blood pressure, *RR* Respiratory rate, *BT* Body temperature, *WBC* White blood cells, *Hb* Hemoglobin, *Plt* Platelet, *LDH* Lactate dehydrogenase, *BUN* Blood urea nitrogen, *K* Serum potassium, *Na* Serum sodium, *ESR* Erythrocyte sedimentation rate, *AST* Enzymes aspartate transaminase, *ALT* Aka alanine aminotransferase, *ALP* Alkaline phosphatase, *FBS* Fasting blood sugar, *BS* Blood sugar, *PaO*_*2*_ Partial pressure of oxygen, *PCO*_*2*_ Partial pressure of carbon dioxide, *HCO3* Bicarbonate, *ICU* Intensive care unit

^a^Data are presented using median (and IQR) except for the following categories (reported as frequency and percentage): sex, HTN, DM, chronic lung disease, treatment group, control group.

**Table 3:** Multivariable regression analysis result for prediction of mortality of patients with severe COVID-19

	Adjusted OR (95% CI)	***P***-value
**IVIg (Treatment Group)**	0.003 (0.001, 0.815)	**0.042**
**Age**	1.485 (1.011, 2.181)	**0.044**
**Systolic BP**	1.078 (0.924, 1.258)	0.336
**Diastolic BP**	0.543 (0.303, 0.972)	**0.040**
**O2 saturation**	0.841 (0.621, 1.138)	0.262
**PLT**	1.000 (0.999, 1.000)	0.132
**LDH**	1.023 (1.000,1.046)	**0.048**
**BUN**	1.136 (0.990,1.304)	0.069
**Creatinine**	0.018 (0.001,6.085)	0.177
**PaO2**	0.834 (0.593,1.173)	0.298

*IVIg* Intravenous immunoglobulin, *BP* Blood pressure, *LDH* Lactate dehydrogenase, *BUN* Blood urea nitrogen, *PaO2* Partial pressure of oxygen

## Discussion

Our results provide evidence to support the administration of IVIg for improving clinical outcomes in COVID-19 patients with severe respiratory system involvement. Prohaska et al. previously conducted research to suggest that IVIg could not be used effectively to reduce the mortality of patients with the acute respiratory distress syndrome (ARDS) undergoing extracorporeal membrane oxygenation (ECMO) therapy [19]. In this study, patients with bacterial and/or fungal infection included 54% of patients in the IVIg treatment group and 28% of patients in the control (placebo) group [19]. As the mechanisms under which our immune system eradicated bacterial and viral infections are not the same, the results of this study could not be generalised for those patients with a merely viral infection. Moreover, a recent randomised control trial by Davey et al. studied the effect of hyperimmune IVIg (hIVIg) on patients with confirmed influenza A and B infections [14]. The study showed that hIVIg was not statistically different from a placebo in treating patients with influenza [20]. The mortality rate of Davey et al. study was 3–4% which is much lower than our study. It is possible that IVIg could be more effective in patients with more severe immune response. Xie et al. recently studied the effect of IVIg treatment timing on mortality rates in patients with critical COVID-19 infection. The study, which included 58 subjects, saw 28 patients die during the 28-day period of admission (mortality = 48.2%). The mortality rate of patients who received IVIg during the first 48 h of admission to the ICU versus those who received IVIg treatment 48 h or more after ICU admission were 23.3 and 57.1%, respectively (*P* = 0.009) [21]. The mortality rates reported by Xie et al. are similar to our findings, suggesting that IVIg treatment, administered early-on, could significantly reduce mortality in critically ill COVID-19 patients. Due to the anti-cytokine effects, inhibition of complement activation, and down-regulation of B and T cells’ functions, IVIg can prevent the excessive body damage when administered at the initial stages of severe infection; therefore, considering this mechanism of action, after permanent tissue damage it would not be effective [22]. Thereupon, early administration of IVIg in server cases of COVID-19 is important [21]. Cao et al. also reported desirable results in the treatment of 3 patients with severe COVID-19 using 25 g/day of IVIg consecutively for 5 days [17]. Our results suggest that IVIg administration of 20 g/day for three consecutive days could be effective and safe in a larger, Iranian population.

Shao et al. conducted a multicenter retrospective cohort study on 325 COVID-19 patients, 222 (68%) with severe COVID-19 and 103 (32%) with critical COVID-19. Among 174 patients, IVIg was administered (treatment group), while 151 patients did not take IVIg (control group). The cohorts had significantly different baseline characteristics. The age of the IVIg group was significantly higher (*p* = 0.009), and in the IVIg group, 41% of patients had the critical type of disease. In the non-IVIg group, the critical type of disease incidence was 21%. The study reported a 28-day mortality rate of 13% among both groups. The primary analysis showed no statistically significant difference between the treatment group and the control group in reducing in-hospital mortality. After adjusting the outcomes of the two groups based on the severity of illness, however, results demonstrated that administration of IVIg did significantly decrease 60-day mortality rates. The same study also showed that both IVIg dosage (> 15 g/d) and administration period (≤ 7 days after hospital admission) could improve efficacy [23].

Our study demonstrated that increasing age, decreasing diastolic blood pressure, and increasing LDH were also statistically associated with higher mortality in COVID-19 patients with severe disease. Correspondingly, Du et al. postulated that a reported age of ≥65 years is associated with higher mortality in patients with COVID-19-related pneumonia [24]. Furthermore, Henry et al. reported that elevated LDH levels are associated with 16-fold higher mortality rates among patients with COVID-19 [25].

To the best of our knowledge, our study is the first randomized placebo-controlled double-blind trial that suggests the effectiveness of IVIg in reducing in-hospital mortality in patients with severe COVID-19 pneumonia. Nonetheless, some limitations affected our study. Research was conducted as a pilot and thus included a relatively small sample size. A further multicenter study with larger samples size should be conducted in this regard. Moreover, the study would be improved if we were to follow patients to assess the intermediate and long-term effects of IVIg treatment on mortality. Due to the current pressing concerns for recommending evidence-based medications for COVID-19 patients, we decided to report follow-up data in future reports. It should also be noted that the cost of IVIg treatment is relatively high, and therefore may not be widely available in World Bank-defined low- and middle-income countries [26].

The patients were consecutively included in our study, and there was no tendency towards including male patients. However, a larger proportion of our sample consisted of male patients. This inequity could be due to the higher prevalence of male sex among COVID-19 cases, which is reported in the majority of studies worldwide [27]. This sex difference is more prominent in Iran (as reported by Nikpouraghdam et al., the male-to-female ratio in COVID-19 cases was 1.93:1 in Iran) [28]. Moreover, severe cases are more prevalent among male patients, and male sex is reported to be an independent predictor of mortality (OR = 1.45, 95% CI: 1.08–1.96) [28]. Nevertheless, there was no significant difference between the treatment and control groups in terms of sex in our study.

Despite the randomized allocation of patients, some parameters were significantly different between the two groups, including WBC, serum creatinine, and duration of hospitalization. Although the difference in creatinine was statistically significant, it was not clinically meaningful. Moreover, the longer duration of hospitalization in the IVIg group could be due to the longer survival of patients in the IVIg group. In other words, those patients with critical status died in the control group, but those with similar critical conditions in the IVIg group survived and stayed longer in the hospital.

## Conclusions

The results of our study suggest that the administration of IVIg in patients with severe COVID-19 infection who did not respond to initial treatments could improve clinical outcomes and thus reduce mortality rates. Regarding high price of IVIg, we suggest that it should be considered in patients with > 30% involvement of lungs in lung CT scan, whom their dyspnea do not improve with standard treatment, those with persistent satO2 under 90%, and those who develop aggravation of lung involvement in serial lung CT scans, especially in younger adults.

## Supplementary information

**Additional file 1: Supplementary Table 1.** Demographic characteristics of patients.

## Data Availability

All Data and material collected during this study are available from the corresponding author upon reasonable request.

## Abbreviations

IVIg: Intravenous immunoglobulin
HR: Heart rate
BP: Blood pressure
RR: Respiratory rate
BT: Body temperature
WBC: White blood cells
HB: Hemoglobin
PLT: Platelet
LDH: Lactate dehydrogenase
BUN: Blood urea nitrogen
K: Serum potassium
Na: Serum sodium
ESR: Erythrocyte sedimentation rate
AST: Enzymes aspartate transaminase
ALT: Aka alanine aminotransferase
ALP: Alkaline phosphatase
FBS: Fasting blood sugar
BS: Blood sugar
PaO_2_: Partial pressure of oxygen
PCO_2_: Partial pressure of carbon dioxide
HCO_3_: Bicarbonate
ICU: Intensive care unit
ECMO: Extracorporeal membrane oxygenation
ARDS: Acute respiratory distress syndrome
CT: Computed tomography
