# Correction to: The use of intravenous immunoglobulin gamma for the treatment of severe coronavirus disease 2019: a randomized placebo-controlled double-blind clinical trial

**DOI:** 10.1186/s12879-020-05628-w

**Published:** 2020-11-26

**Authors:** Naser Gharebaghi, Rahim Nejadrahim, Seyed Jalil Mousavi, Seyyed-Reza Sadat-Ebrahimi, Reza Hajizadeh

**Affiliations:** 1 Department of Infectious Diseases, Urmia University of Medical Sciences, Urmia, Iran; 2 Cardiovascular Research Center, Tabriz University of Medical Sciences, Tabriz, Iran; 3 Department of Cardiology, Urmia University of Medical Sciences, Urmia, Iran

**Correction to: BMC Infect Dis (2020) 20:786**

**https://doi.org/10.1186/s12879-020-05507-4**

Following publication of the original article [1], the author identified two errors in the Results section of the Abstract and in Table 2.

The sentence currently reads:

“ The in-hospital mortality rate was significantly lower in the IVIg group compared to the control group (6 [20.0%] vs. 14 [48.3%], respectively; P = 0.022). “.

The sentence should read:

“ The in-hospital mortality rate was significantly lower in the IVIg group compared to the control group (6 [20.0%] vs. 14 [48.3%], respectively; P = 0.025). “.

Furthermore, the correct Table 2 is given below.

**Table 2:** The relationship between study variables and mortality of patients with severe COVID-19

		Mortality		Unadjusted OR (95% CI)	***P***-value
		No	Yes		
**Treatment Group**		24 (61.5)	6 (30)	0.27 (0.08, 0.85)	**0.025**^**#**^
**Control Group**		15 (38.5)	14 (70)	Ref	
**Age (years)**		54 (44, 60)	60 (53.5, 70)	1.05 (1.01, 1.1)	**0.014**
**Gender n (%)**	**Male**	27 (70)	14 (70)	0.96 (0.3, 3.12)	0.951
	**Female**	12 (30)	6 (30)	Ref	
**HTN n (%)**		8 (23.3)	5 (25)	1.29 (0.36, 4.63)	0.694
**DM n (%)**		11 (20)	5 (25)	0.85 (0.25, 2.9)	0.793
**Chronic lung disease n (%)**		1 (6.6)	1 (5)	2 (0.12, 33.76)	0.630
**HR/min**		95 (90, 100)	95 (89, 108)	1.02 (0.96, 1.08)	0.543
**Systolic BP (mmHg)**		120 (120, 130)	120 (110, 130)	0.97 (0.93, 1.01)	**0.173**
**Diastolic BP (mmHg)**		80 (70, 80)	70 (70, 80)	0.95 (0.88, 1.01)	**0.120**
**RR /min**		19 (18, 22)	20 (18, 22)	0.98 (0.93, 1.04)	0.545
**BT C°**		37.1 (36.7, 37.7)	37.1 (36.75, 37.65)	1.26 (0.63, 2.52)	0.518
O_2_ **saturation (%)**		89 (87, 89)	85 (80, 88)	0.84 (0.74, 0.97)	**0.015**
**WBC (1000/mm**^**3**^**)**		5.1 (4.6, 7.4)	7.2 (5.1, 11.2)	1.00 (1.00, 1.00)	0.743
**Hb (g/dl)**		13.9 (12.6, 15)	13.5 (12.05, 15.25)	0.95 (0.8, 1.13)	0.566
**Plt (1000/mm**^**3**^**)**		210 (135, 247)	172.5 (139.5, 193)	1.00 (1.00, 1.00)	**0.090**
**LDH (U/L)**		520 (400, 687)	761 (560.5, 1021)	1.00 (1.00, 1.00)	**0.003**
**BUN (mg/dl)**		29 (25, 39)	64 (50, 129.5)	1.06 (1.02, 1.09)	**0.001**
**Creatinine (mg/dl)**		1 (1, 1.2)	1.2 (1.1, 1.55)	3.63 (0.95, 13.87)	**0.059**
**K (mEq/L)**		4 (3.7, 4.4)	4.2 (4.1, 4.3)	0.95 (0.8, 1.14)	0.616
**Na (mEq/L)**		138 (137, 139)	138 (135, 143.5)	1.04 (0.95, 1.14)	0.344
**ESR**		28 (20, 41)	40 (26, 49.5)	1.01 (0.99, 1.03)	0.424
**AST (U/L)**		31 (21, 39)	29.5 (15.5, 52.5)	1.00 (0.97, 1.02)	0.735
**ALT (U/L)**		34.5 (25, 41)	38.5 (29.5, 48)	1.01 (0.99, 1.02)	0.306
**BS (mg/dl)**		119 (105, 174)	126 (109.5, 184)	1.00 (0.99, 1.01)	0.845
**Pa**O_2_ **(mmHg)**		46 (40, 50)	39.5 (34, 49)	0.92 (0.85, 0.99)	**0.035**
**PC**O_2_ **(mmHg)**		40 (35, 46)	38 (36, 39.5)	0.99 (0.91, 1.08)	0.798
**HCO**_**3**_ **(mEq/L)**		24 (22, 25)	24.5 (19.5, 26.5)	1.00 (0.89, 1.13)	0.946
**pH**		7.3 (7.3, 7.4)	7.3 (7.2, 7.4)	0.04 (0, 8.87)	0.243

The original article [1] has been corrected.
