# Supplementary material for: The use of intravenous immunoglobulin gamma for the treatment of severe coronavirus disease 2019: a randomized placebo-controlled double-blind clinical trial
Source: BMC Infect Dis. 2020 Oct 21;20:786. doi: 10.1186/s12879-020-05507-4 (PMC7576972; doi:10.1186/s12879-020-05507-4)
Supplement: Supplementary file 1 — Additional file 1: Supplementary Table 1. Demographic characteristics of patients. [file 12879_2020_5507_MOESM1_ESM.docx]

**Supplementary Table 1. Demographic characteristics of patients**

|  | | **Total** | ***IVIg Group*** | **Control Group** | ***P*-value** |
| --- | --- | --- | --- | --- | --- |
| **Age (years)*** |  | 56 (46 ,62) | 55.5 (45 ,60) | 56 (47 ,66) | 0.375 |
| **Sex n (%)** | **Male** | 41 (69.5) | 21 (70) | 20 (68.9) | 0.931 |
|  | **Female** | 18 (30.5) | 9 (30) | 9 (31) |  |
| **Residence** | **Rural** | 18 (30.5) | 10 (33.3) | 8 (27.5) | 0.632 |
|  | **Urban** | 41 (69.5) | 20 (66/7) | 21 (72/5) |  |
| **Race** | **Iranian** | 59 (100) | 30 (100) | 29 (100) | 1.000 |
| **Occupation** | **Medical** | 5 (8.5) | 3 (30) | 2 (6.9) | 0.669 |
|  | **Non-medical** | 54 (91.5) | 27 (90) | 27 (93.1) |  |
